# Case report: Spontaneous carotid-cavernous fistula associated with persistent primitive trigeminal artery aneurysm rupture

**DOI:** 10.3389/fneur.2022.923186

**Published:** 2022-09-06

**Authors:** Peng Sun, Yuan Chai, Wei Fang, Hu Chen, Qianfa Long, Zhenwei Zhao, Tao Zhang

**Affiliations:** ^1^Department of Neurosurgery, Tangdu Hospital, Air Force Medical University, Xi'an, China; ^2^Department of Neurosurgery, Xi'an Central Hospital, Xi'an, China

**Keywords:** spontaneous carotid-cavernous fistula, persistent primitive trigeminal artery, aneurysm, interventional treatment, case report

## Abstract

**Background:**

The incidence of carotid cavernous fistula (CCF) associated with persistent primitive trigeminal artery (PPTA) aneurysm rupture is extremely rare. We presented a case about a spontaneous CCF secondary to a ruptured PPTA aneurysm, which was successfully embolized with coils and onyx-18 by a trans-arterial approach.

**Case presentation:**

A 55-year-old female suffered a sudden onset of headache, left orbital pain, and pulsatile exophthalmos for a month without any history of trauma. Angiography revealed a left-sided CCF associated with a ruptured PPTA aneurysm, with major drainage to the ipsilateral superior ophthalmic vein. Through a trans-arterial approach, the fistula and ruptured PPTA aneurysm were embolized with coils and onyx-18, while the cavernous sinus and PPTA were well-preserved. However, the preserved PPTA vanished at 4 month follow-up. The patient had no neurological deficit from hospitalization to 1 year follow-up period.

**Conclusion:**

Trans-arterial approach was a reasonable choice for spontaneous CCF associated with ruptured PPTA aneurysm. The requirement for PPTA preservation depended on individual evaluation.

## Introduction

Persistent primitive trigeminal artery (PPTA) is the most common embryonic communication between vertebra-basilar and carotid systems in adulthood. It has been reported to occur in 0.1–1% of the population (1). The origin of PPTA is usually in the posterior or lateral surface of the intracavernous internal carotid artery (ICA), proximal to the origin of the meningo-hypophyseal trunk (MHT). The PPTA passes in sequence through the cavernous sinus (CS) and subarachnoid space, then enters into the basilar artery (BA) between the superior cerebellar artery (SCA) and anterior inferior cerebellar artery (AICA) (2). PPTA can be associated with many vascular anomalies and disorders, including aneurysm, arteriovenous malformation (AVM), and carotid cavernous fistula (CCF). Due to its anatomical features, a ruptured PPTA aneurysm may lead to CCF or subarachnoid hemorrhage (SAH) (3). The incidence of CCF associated with PPTA aneurysm rupture is extremely rare. We presented a case about a spontaneous CCF secondary to a ruptured PPTA aneurysm, which was successfully embolized with coils and onyx-18 by a trans-arterial approach.

## Case presentation

**Figure 1:**
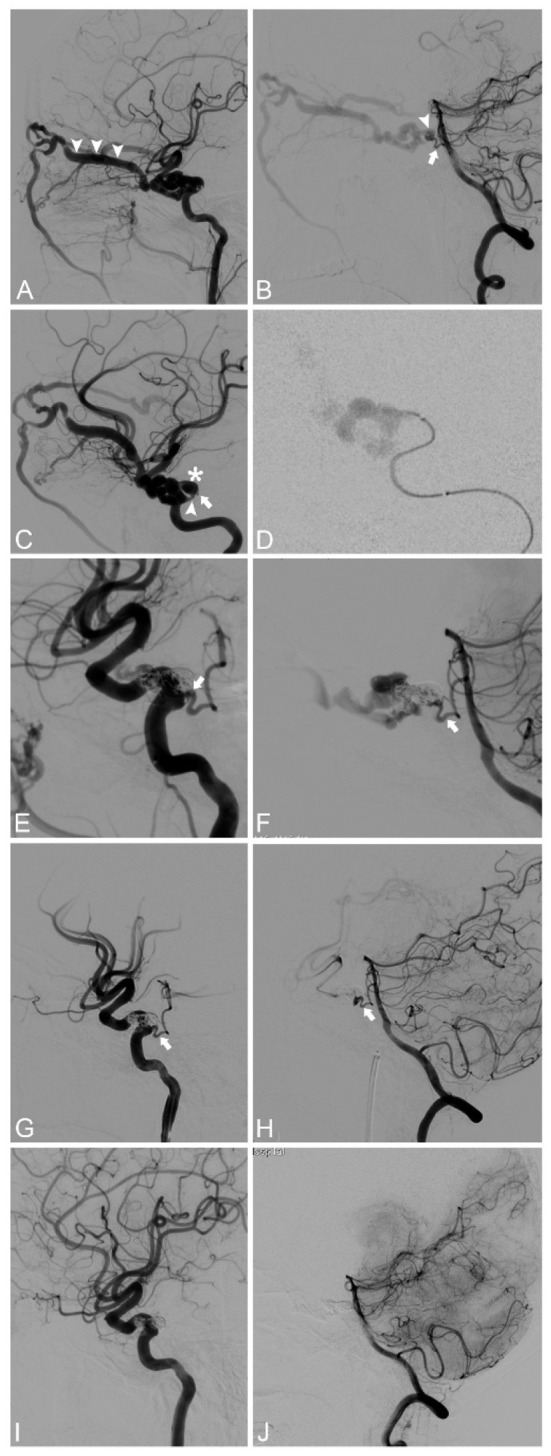
**(A)** Lateral view of the left ICA angiography showing a CCF with dilated SOV (arrowhead). **(B)** Lateral view of the right VA angiography showing the retrograde blood flow (arrow) from a ruptured PPTA aneurysm (arrowhead), projecting into the left CS. **(C)** The optimal projection for PPTA emitting (arrowhead) from left ICA, the PPTA aneurysm (asterisk), and the distal orifice (arrow) of PPTA connecting to BA. **(D)** The fistula was confirmed by super-selective angiography. **(E,F)** Partial embolization of the fistula and the adjacent PPTA aneurysm exhibited the PPTA trajectory (arrow) more evident than untreated. **(G,H)** The fistula and aneurysm were completely embolized, while the CS and PPTA (arrow) were well-preserved. **(I,J)** Four-month follow-up angiography demonstrated complete obliteration of the fistula and PPTA aneurysm, while the preserved PPTA vanished. BA, Basal Artery; CCF, Carotid Cavernous Fistula; CS, Cavernous Sinus; ICA, Internal Carotid Artery; PPTA, Persistent Primitive Trigeminal Artery; SOV, Superior Ophthalmic Vein; VA, Vertebral Artery.

A 55-year-old female of Chinese origin presented with sudden onset of headache and left orbital pain without any history of head trauma a month ago. She also suffered from progressive left-sided exophthalmos and diplopia since then. Physical examination on admission demonstrated left-sided pulsatile orbital bruit, chemosis, and abducens nerve paresis (Table 1). This patient had a history of surgery for femoral shaft fracture 3 years ago, but it was decided this was probably irrelevant to the present complaint. Computed tomography showed an enlarged left superior ophthalmic vein (SOV). Left ICA angiography revealed a left-sided CCF associated with a ruptured PPTA aneurysm, with major drainage to the ipsilateral SOV (Figure 1A). Vertebral angiography also revealed a retrograde blood flow of PPTA from BA to CS (Figure 1B).

**Table 1:** Timeline of clinical and procedural data.

**Admission**	**Spontaneous headache, left orbital pain and pulsatile exophthalmos**
Day 3	PE: Left orbital bruit, chemosis and abducens nerve paresis
Day 4	Cerebral angiography
Day 5	Embolized with coils and onyx-18 by trans-arterial approach
Day 10	Discharged without any neurological deficit during hospitalization
4 month	Clinical and angiographic follow up
1 year	Clinical follow up

PE, physical examination.

Endovascular embolization with detachable coils and Onyx-18 liquid embolic system (Medtronic-ev3, Minneapolis, MN, USA) by a transarterial approach was planned. Under general anesthesia, 4,000 units of heparin were intravenously administered to the patient. A 6F Envoy guiding catheter (Codman & Shurtleff, Inc., Raynham, MA, USA) was introduced through a femoral sheath into the petrous segment of the left ICA. Under an optimal projection for PPTA emitting from left ICA (Figure 1C), an Echelon-10 microcatheter (Medtronic-ev3, Minneapolis, MN, USA) guided by a Synchro2 microguidewire (Stryker, Salt Lake, Utah, USA) was directed into the PPTA aneurysm which was confirmed by super-selective angiography (Figure 1D). Partial embolization of fistula and proximal ruptured PPTA aneurysm exhibited the PPTA trajectory more obviously than untreated (Figures 1E,F). Finally, the fistula and aneurysm were completely embolized with four Axium detachable coils (Medtronic-PLC, Dublin, Ireland) followed by a 1.6 ml Onyx-18 liquid embolic system, while the CS and PPTA were well-preserved (Figures 1G,H).

The left orbital bruit and chemosis of the patient subsided immediately after embolization, while residul mild abducens nerve paresis lasted for a month. There is no focal neurological deficit during hospitalization. Four-month follow-up angiography demonstrated complete obliteration of the fistula and PPTA aneurysm. However, the preserved PPTA vanished (Figures 1I,J), but the patient had no neurological deficit. No evidence of recurrence was observed during the 1-year clinical follow-up.

## Discussion

### Embryology

At 28–29 days of embryonic development (3 mm human embryonic stage), there are four special channels, including the proatlantal artery, hypoglossal artery, otic artery and trigeminal artery, which are connecting the carotid systems with the longitudinal neural arteries (4). At the 14 mm human embryonic stage, the posterior communicating artery (PComA) replaces the previous four vessel channels, which regress and are subsequently obliterated as the major blood supply to the posterior circulation (4). The PPTA is the largest and most common fetal carotid-basilar anastomotic artery that persists into adulthood, although the exact cause for its persistence remains unclear.

### Etiology

Despite its extremely rare incidence, PPTA can be associated with aneurysms, AVMs, and CCF. Due to the trajectory of the PPTA, spontaneous ruptured PPTA aneurysm may cause CCF or SAH. The CCF associated with the PPTA may also be traumatic. When an external force acts on the head in trauma, the PPTA, as the most susceptible target, bears maximum shear stress during acceleration or deceleration injury (3, 5). The tear in the proximal segment of the PPTA results in traumatic CCF. However, owing to the structural vulnerability of the PPTA, formation and subsequent rupture of PPTA aneurysm often lead to spontaneous CCF (3). Furthermore, delayed rupture of the dissecting PPTA aneurysm resulting from trauma may also be the cause of spontaneous CCF (6).

In our case, a spontaneous CCF was identified by angiography in a patient without trauma history. The root of the PPTA (Figure 1C, arrowhead) emitted from the left cavernous ICA and then poured into an associated aneurysm (Figure 1C, asterisk) which was ruptured in the cavernous sinus to form a CCF. The orifice of the distal PPTA connecting to the BA was also noticed (Figure 1C, arrow). For hemodynamic reasons, the direction of blood flow in the distal PPTA reversed from the BA to the cavernous sinus during VA angiography (Figure 1B). Super-selective angiography confirmed the fistula and associated PPTA aneurysm (Figure 1D).

### Classification

Based on its emitting site in the ICA and vascular course, the PPTA may be classified into lateral or medial subtype, both of which are equally common in the population (7). In the lateral subtype, the artery arose from the posterolateral portion of the intracavernous ICA. It courses adjacent to the lateral wall of the CS. When crossing the CS, the PPTA passes between the abducens nerve and the ophthalmic division of the trigeminal nerve. Conversely, when the PPTA arises from the posteromedial aspect of the cavernous segment of the ICA, it should be medial to the abducens nerve in the majority of cases (8). Along its intracavernous course, the PPTA may give rise to the branches to the trigeminal nerve and the pituitary (9).

In 1959, Saltzman reported eight cases about the PPTA, and proposed an angiographic classification (10, 11), In Saltzman type 1, the PPTA inserts into the BA, between SCA and AICA. The BA, proximal to the PPTA insertion, may be hypoplastic and the PComA may be absent or poorly developed. In other words, the main blood supply for the distal BA, PCA, and SCA comes from the PPTA, thus the PPTA must be well-preserved in order to avoid posterior circulation ischemia during treatment (10, 11). In Saltzman type 2, the PPTA inserts into the BA before the origin of SCA, but only supplies the bilateral SCAs, while the PCAs are supplied by the PComA (10, 11). Later, both Saltzman and Parkinson demonstrated cases as a combination variant of Saltzman type I and II (12, 13). This means the PPTA supplied the bilateral SCAs and contralateral PCA, while a fetal PComA supplies the ipsilateral PCA. Many Saltzman variants, referred to as Saltzman type 3, have been reported subsequently. The PPTA variants terminated directly to the SCA (Saltzman Type 3a), AICA (Saltzman Type3b), or PICA (Saltzman Type3c) rather than inserting into the BA (4).

The PPTA in our case inserted into the BA between SCA and AICA, but the BA proximal to the PPTA insertion was well-developed and provided sufficient blood supply for bilateral PCAs and SCAs. Thus, the PPTA could be safely sacrificed when treating the PPTA aneurysm and the associated CCF.

### Treatment

Treatment strategies for PPTA aneurysm associated with CCF involve the occlusion of the fistula pouch and adjacent ruptured PPTA aneurysm while minimizing the impact on normal arterial supply and venous drainage (3). In 1977, Enomoto et al. reported a spontaneous CCF induced by a ruptured PPTA aneurysm. The patient's ICA was then ligated without any signs of ischemia due to the inverted blood flow of the PPTA (14). Due to the low technique success rate and inevitable complications, microsurgery had been replaced by endovascular treatment. In 1982, Charlin and his colleagues treated a PPTA aneurysm-associated CCF with a detachable balloon (15). However, the detachable balloon could only be applied for selected cases because of its inherent material limitation (16).

In 1998, Bernstein et al. treated a spontaneous CCF associated with a ruptured PPTA aneurysm with coils for the first time (17). In 2008, Geibprasert et al. performed a staged treatment for the same disease. Firstly, they coiled the fistula with 19 coils through the right SOV by a trans-venous approach. Then, they embolized the remnant with N-butyl cyanoacrylate (NBCA) through the right PPTA by a trans-arterial approach. It was the first time that a liquid embolic agent had ever been used for such diseases (3). Since then, coiling with liquid embolic agent became the first choice for such types of CCF cases.

Both trans-arterial and trans-venous approaches could be applied for the treatment according to individual angioarchitecture. When the trans-arterial approach is too tortuous and a microcatheter could not be successfully guided into the targeted position, the trans-venous approach could then be adopted (18). However, trans-venous embolization was believed to require more coils or liquid embolic agent than trans-arterial embolization. The mass effect of excessive cavernous sinus packing might cause iatrogenic cranial nerve palsy and other complications. Moreover, unexpected obliteration of the anatomical drainage from the CS may lead to venous hypertension. Subsequently, the blood would reflux into the cortical or ophthalmic veins, which would increase the risk of hemorrhage or aggravate ocular symptoms (19). In 2018, Imrie et al. treated a spontaneous CCF secondary to a ruptured PPTA aneurysm with detachable coils in the CS through the inferior petrosal sinus (IPS). Eventually, the CS was completely occluded and the fistula vanished (20). In 2000, Oka et al. also used detachable coils to treat a similar CCF through the right IPS approach. Although, the fistula wasn't completely occluded immediately after treatment. Fourteen days later, follow-up angiography revealed the complete disappearance of the fistula probably due to thrombosis, which was presumably accelerated by the coils (19).

Compared with trans-venous embolization, trans-arterial embolization was believed to be a more reasonable choice for treating the PPTA aneurysm-associated CCF, due to the short access in distance and minor mass effect (21). Through the trans-arterial approach, a microcatheter could be navigated into the fistula pouch within the CS through the ruptured PPTA aneurysm. Thus, targeted embolization for the fistula pouch and ruptured aneurysm could be achieved, while the structure and function of the CS could be well-preserved (22). In 2011, Yoshida et al. treated a PPTA aneurysm associated CCF with coils. They failed to navigate the microcatheter into the fistula pouch by a transvenous approach because the fistula orifice was too small. Then they embolized the PPTA aneurysm with detachable coils by a trans-arterial approach. Finally, the fistula disappeared (23). In 2019, Fan et al. navigated a microcatheter into the CS through the PPTA aneurysm crevasse by a trans-arterial approach. The CS was firstly carefully embolized with detachable coils. After ipsilateral ICA protection with a Hyper-Glide balloon, the fistula and PPTA aneurysm was then embolized with Onyx-18 (18).

As for specific PPTA aneurysm-associated CCF cases, both trans-arterial and trans-venous approaches had to be used together for treatment. Taichi et al. successfully treated a spontaneous case of CCF secondary to a PPTA aneurysm with a multipronged embolization strategy, which meant a combination of trans-arterial with a trans-venous approach. Three microcatheters were guided into the fistula as follows: (1) through the PPTA to the CS from the left ICA, (2) through the PPTA to the CS from the BA, and (3) through the CS to the PPTA aneurysm from the internal jugular vein. The fistula was then completely occluded without any signs of recurrence during the follow-up period. They suggested that this multipronged approach was safe and effective for tortuous artery cases and was beneficial for avoiding incomplete fistula occlusion (24).

Whether the PPTA should be preserved or not is another concern for treatment strategies. In Saltzman type 1, the PPTA is a critical blood supply for the posterior circulation, thus PPTA must be retained (10, 11). In Saltzman type 2, the blood flow of the PCA mainly comes from the PComA. It seems that PPTA preservation is not so critical. However, as we have mentioned before, the PPTA may give off branches to the trigeminal nerve, pituitary, and brainstem (9–11). Thus, the sacrifice of the PPTA needs cautiously evaluation.

As to our case, because the PPTA was not too tortuous, there was no difficulty in navigating the microcatheter into the PPTA aneurysm and subsequent fistula pouch through a trans-arterial approach. Our concern focused on the following points: (1) maximizing the preservation of the CS structure and function, (2) whether the PPTA could be sacrificed or not, and (3) the possibility of onyx-18 reflux into the ICA or BA. As we noticed that the bilateral VAs were well-developed and there was inverted blood flow in the PPTA from BA to the fistula. We were confident that it was quite safe to sacrifice the PPTA. Even so, we decided to try our best to preserve of the PPTA during treatment. On account of the enough PPTA reflux distance and unnecessary PPTA preservation, balloon protection for the ICA or BA was not needed. Firstly, we cautiously coiled the very regional fistula pouch and proximal PPTA aneurysm with proper size coils as a framework. Onyx-18 was then carefully injected within the coil framework as far as we possibly could until complete fistula occlusion. Finally the CS was minimally sacrificed and the PPTA was well-preserved. This patient recovered well without any ischemic neurological deficit during hospitalization. Four months later, follow-up angiography revealed the disappearance of the well-preserved PPTA, probably owing to the delayed PPTA occlusion, but the patient encountered no related symptom.

## Conclusion

The incidence of spontaneous CCF secondary to the PPTA aneurysm rupture is extremely rare. Trans-arterial embolization by coils with a liquid embolic agent is a safe and effective treatment for selected cases. Preservation of the PPTA depends on the individual assessment.

## Data availability statement

The original contributions presented in the study are included in the article/supplementary material, further inquiries can be directed to the corresponding author/s.

## Ethics statement

Written informed consent was obtained from the individual for the publication of any potentially identifiable images or data included in this article. The Ethics Committee of Tangdu Hospital of Fourth Military Medical University approved the research protocol in compliance with the Helsinki Declaration.

## Author contributions

TZ and YC completed the operation. WF and HC assisted the operation. PS wrote the first draft of the manuscript. QL and ZZ reviewed the case and manuscript. TZ designed the treatment strategy and final revised the manuscript. All authors contributed to the article and approved the submitted version.

## Conflict of interest

The authors declare that the research was conducted in the absence of any commercial or financial relationships that could be construed as a potential conflict of interest.

## Publisher's note

All claims expressed in this article are solely those of the authors and do not necessarily represent those of their affiliated organizations, or those of the publisher, the editors and the reviewers. Any product that may be evaluated in this article, or claim that may be made by its manufacturer, is not guaranteed or endorsed by the publisher.
